# Physician’s knowledge and opinions on human papillomavirus vaccination: a cross-sectional study, Saudi Arabia

**DOI:** 10.1186/s12913-019-4756-z

**Published:** 2019-12-12

**Authors:** Nisreen M. Anfinan

**Affiliations:** Gynecology Oncology Unit, Department of Obstetrics and Gynaecology, Faculty of Medicine, King Abdulaziz University, Jeddah, 21589 Saudi Arabia

**Keywords:** Human papilloma virus, Vaccine, Vaccination, Awareness, Knowledge, Attitude, Saudi Arabia, Physicians, Prevention

## Abstract

**Background:**

In a transition period of prevention strategy against HPV infection and cervical cancer in Saudi Arabia, it becomes necessary to appraise physicians’ preparedness to undertake the inherent actions and responsibilities, by evaluating their knowledge and opinions regarding HPV infection and vaccine.

**Methods:**

A cross-sectional study carried out between Jan 2017 and Nov 2018, included 2000 physicians working in 21 public centers from the five regions of Saudi Arabia. A self-administered questionnaire was used to assess physicians’ perception about HPV infection prevalence (1 item), knowledge about HPV infection and vaccine (9 items), and opinions and attitudes toward vaccine (4 items). A knowledge score (range 0–9) was calculated and adequate knowledge was assumed for a score ≥ median. Factors associated with opinions and attitudes were explored and multivariate regression was used to analyze independent factors of inadequate knowledge (score < median).

**Results:**

Majority of the participants replied correctly to all knowledge questions, and 63.0% perceived HPV infection as a frequently encountered infection. Median knowledge score was 8 and 62.0% had adequate knowledge (score ≥ 8). Inadequate knowledge was independently associated with Saudi nationality (OR = 1.51, *p* = 0.003), practice level (resident: OR = 3.53, *p* < 0.001; junior OR = 1.67, *p* = 0.002), and non Ob-Gyne specialty (OR = 5.40, *p* < 0.001); in addition to disparities across region and age. Among the participants, 7.6% were immunized and 41.2% accepted to receive the vaccine, while majority were favorable to have their children vaccinated (77.6%) and to include HPV vaccine in the local immunization program (69.6%). Self-perceived underexposure to HPV infection (58.5%), lack of knowledge about the vaccine (21.1%) and being sexually inactive (14.7%) were the most frequently reported reasons for refusing the vaccine. Overall negative attitude regarding vaccine was typically associated with male, older, Saudi, senior consultant in other than Ob/Gyn specialty. Inadequate knowledge level as well as lowly perceived prevalence of HPV infection were associated with less favorable attitude to vaccination.

**Conclusions:**

More specific educational interventions are warranted to trigger physicians’ active engagement in the fight against HPV infection and cervical cancer. Such interventions should demystify the HPV vaccine by exposing its efficacy, availability and safety, along with providing practical information about the vaccination procedure and goals to achieve successful prevention strategy.

## Background

Human papilloma viruses (HPVs) are considered common pathogens that are disseminated through sexual intercourse. Several genotypes of HPV were identified, which are classified into low- or high-risk depending on their oncogenic potential on the infected cells. Low-risk genotypes are generally associated with anogenital warts, while high-risk ones are associated with malignant tumors of the genital tract, notably cervical cancers and, to a lesser extent, endometrial and ovarian cancers [1–4].

Global observations suggest that 75% of sexually active adults would contract an HPV infection during their lifetime [5]. However, the incidence depends on several factors such as age, risky sexual behavior, and cultural norms [6]. Consequently, HPV prevalence is relatively low among conservative cultures such as Saudi Arabia, where sexual relationships are committed to strict social and religious rules. However, an alarming increase in the incidence of HPV infections is reported in the last decade in Saudi Arabia, where, despite the conflicting results, up to 43% of cervical samples among healthy Saudi women are reported to be HPV DNA-positive [6–10]. These data highlight a change in the epidemic trend of HPV in Saudi Arabia, besides a lack of reliable information to provide an accurate epidemiological picture.

The major threat of HPV infections remains the cervical cancer among infected women. Cervix cancers ranked fifth most fatal and fourth most common cancers worldwide by the early 2010’s [11]. They occur most frequently between the age of 15 and 44 years [12]. In Saudi Arabia, cervical cancers ranked eighth among the most common cancers in Saudi females [4, 13], and an HPV infection is incriminated in 89–96% of cases, with HPV-16 and 18 being the most common genotypes [14, 15].

Therefore, the globally adopted strategy in cervical cancer prevention includes systematic HPV cervical screenings and vaccination against HPV. Majority of the developed countries recommended preventive vaccination against HPV as a routine vaccine for 11–12 year-old population. As well, the vaccine was recommended for unvaccinated females and males who were between 13 and 26 and 13–21, respectively [13]. Vaccination campaigns showed prodigious and rapid efficacy in reducing the incidence of genital warts and cervix cytological abnormalities; thus, providing additional arguments to reinforce vaccine recommendations and set new strategic goals to control the HPV related diseases [16, 17].

However, the implementation of such strategies is slow to appear in several developing countries, which is thought to explain 85% of the global burden of mortality from cervical cancer today [4, 6, 18–20]. Among the factors challenging the implementation of such HPV vaccination programs are inadequate finance, competition with compulsory vaccines, and shortage of valid statistics on the burden of HPV diseases. In Saudi Arabia, in spite of the availability of the vaccine, no HPV vaccination program is introduced at the national level to date and the vaccination rates are very low [4, 19].

On the other hand, raising awareness among the general population as well as among specific high-risk subpopulations is considered the most effective method to prevent HPV infection. This requires active involvement by the physicians, and specifically the obstetric gynecology, family medicine and primary health care physicians, to promote the vaccine and other preventive measures among the community [21, 22]. However, inadequate knowledge and awareness among physicians about the importance of the HPV vaccine was highlighted as one of the major limitations to successful vaccination programs [23, 24]. Therefore, we attempted to assess the level of knowledge and perception among physicians from Saudi Arabia regarding HPV infection and vaccine, as well as their opinion and attitude towards individual and systematic vaccinations.

## Methods

### Design & population

This cross-sectional study involved physicians from all regions of the Kingdom of Saudi Arabia, and was conducted between January 2017 and June 2018. Eligibility criteria applied for both gender, all nationality residents and junior and senior physicians, who were qualified by the Saudi Commission for Health Specialties and currently working at a governmental hospital or clinic in Saudi Arabia. Physicians who were employed as part time or locum, as well as those who were on leave at the time of data collection were excluded. The study was approved by the Institutional Review Board of the Saudi Ministry of National Guard Health Affairs (MNG-HA) and the committee board of the Saudi Ministry of Health (MOH).

### Sample size

Sample size was calculated to detect an unknown percentage (*P* = 50%) of physicians with adequate knowledge, with ±0.03 precision, 80% statistical power and 95% confidence level, among a target population of 10,641 physicians working in Saudi Arabia as estimated in 2017 [25]. The calculation used the formula: n = (Z^2^ * P(1 - P))/e^2^.

where:

- Z = value from standard normal distribution corresponding to desired confidence level (Z = 1.96 for 95% confidence interval [CI]),
- P is expected true proportion = 0.5, as true proportion unknown,
- e is desired precision = 0.03 (half desired CI [0.47–0.53] width).

The sample size was calculated as *n* = 971, which was doubled (*n* = 1942) and up rounded to *n* = 2000 to enable more powerful subgroup analysis.

### Sampling technique

A stratified clustered sampling was used to randomly select a number of clinics (clusters) from each of the five regions (strata) of the Kingdom. The number of clinics by region was proportional to the number of big cities and to the total number of clinics as follows: Eastern (Al-Dammam city, 3 clinics), Western (Tabuk, Al-Madinah and Makkah cities, 10 clinics), Northern (Arar, Sakakah and Hail cities, 3 clinics), Southern (Abha, Najran, Al-Bahah and Jizan cities, 4 clinics) and Central region (Riyadh city, 3 clinics). Thus, a total 21 clinics were selected, expecting approximately 100 participations from each clinic to fulfil the target sample size (*n* = 2000).

### Data collection

A self-administered questionnaire was designed for the purpose of this study; it was redacted in English language and constructed in three sections (Additional file 1). Section 1 covered the demographic characteristics including gender, age, nationality (Saudi versus non-Saudi), marital status, level of practice (resident, junior physician, and senior consultant), specialty and region. Section Two assessed physicians’ knowledge about HPV infection, screening and vaccination, such as whether HPV is sexually transmitted, whether it can affect males, females or both genders, and whether vaccine protects against all HPV serotypes, etc. (9 items). Additionally, a question about physician’s perception about the epidemiological extent of HPV infection worldwide (whether it is frequent or not) was added to Section Two. Section three explored physicians’ opinions and attitudes towards HPV vaccination including whether they have already been vaccinated, their agreement to be vaccinated or to have their children / future children vaccinated, and whether they are favorable to HPV vaccine inclusion in the Saudi immunization program (4 items). Additionally, reasons for eventual refusal of HPV vaccination (for self or for own children) were explored using a multi-response sub-questionnaire that included 6 optional reasons for refusal such as “I’m not under risk for HPV infection”, “lack of knowledge about the vaccine”, “vaccine side effects”, etc.

The questionnaire underwent face and content validity by a group of four Obstetrics & Gynecology consultants, who assessed the relevance and accuracy of each item and provided remarks and suggestions where necessary. A final version was edited and pre-tested for feasibility, showing that less than 10 min were required to fill the questionnaire. Subsequently, hard copies of the questionnaire were printed and attached to an informed consent, and enclosed in an anonymous envelope. Envelopes were delivered for the heads of the participating centers; each head was conveyed to randomly distribute the envelopes to physicians working in his or her center. The consent of the participants was obtained by voluntarily signing at the end of the questionnaire. A total 3600 envelopes were delivered to the heads of the centers.

### Statistical analysis

Statistical analysis was performed with the Statistical Package for Social Sciences version 21.0 for Windows (SPSS Inc., Chicago, IL, USA). Descriptive statistics were used to summarize participant’s charactereistics and patterns of answers to knowledge and opinion and attitude items. A knowledge score (range = 0–9) was calculated as the sum of correct answers given by the participant regarding the 9 knowledge questions. Analysis of knowledge score distribution using Kolmogorov-Smirnov statistics (0.215, *p* < 0.001) and Shapiro-Wilk statistics (0.217, *p* < 0.001) indicated non normal distribution of the variable. Consequently, factors associated with knowledge were analyzed using two methods: 1) nonparametric tests (Mann-Whitney U test and Kruskal-Wallis test, as appropriate) to compare raw knowledge scores between different factor’s categories; 2) chi-squared test to compare the percentage of participants with adequate knowledge level, defined as knowledge score ≥ median value, between the factor’s categories. Results are presented as mean, median and range for nonparametric tests and frequency and percentage of individuals for chi-squared test. A multivariate binomial regression model was carried out to analyze independent factors of inadequate knowledge, defined as knowledge score < median value. Further, chi-square test was used to analyze the association of opinions and attitudes regarding HPV vaccination with demographic factors as well as with knowledge level. A *p* value of < 0.05 was considered to reject the null hypothesis.

## Results

### Participants’ characteristics

Two thousand completed questionnaires were retrieved, out of the 3600 distributed (response rate = 55.6%). Participants’ demographic characteristics showed that more than half were females (52.8%), two-third were Saudi (67.0%), and more than three-quarter were aged 20–50 years (17.6% aged 20–30, 30.4% aged 31–40, and 25.4% aged 41–50) and married (79.4%). Professional characteristics showed majority of senior (50.2%) and Obstetrics-Gynecology (58.6%) consultants. Distribution by region showed highest percentage of Western region (57.4%), followed by Eastern and Central regions with 15.4% each (Table 1).

**Table 1:** Demographic characteristics of the participants

Parameter / category	Number (*N* = 2000)	Percentage
Gender		
Female	1056	52.8
Male	944	47.2
Nationality		
Saudi	1340	67.0
Others	660	33.0
Age groups		
20–30	352	17.6
31–40	608	30.4
41–50	508	25.4
51–60	420	21.0
Above 60	112	5.6
Marital status		
Single	268	13.4
Married	1588	79.4
Widowed	20	1.0
Divorced	124	6.2
Level of practice		
Resident	500	25
Junior	496	24.8
Senior	1004	50.2
Specialty		
Oby/gyn	1172	58.6
Non oby/gyn	818	42.4
Family Medicine	128	6.4
Medicine Radiology	336	16.8
Surgery / Anesthesia / Pathology	296	14.8
Pediatrics	52	2.6
Dentistry	16	0.8
Region		
Western	1148	57.4
Eastern	308	15.4
Northern	116	5.8
Southern	116	5.8
Central	308	15.4

### Knowledge about HPV infection and vaccination

**Fig. 1:**
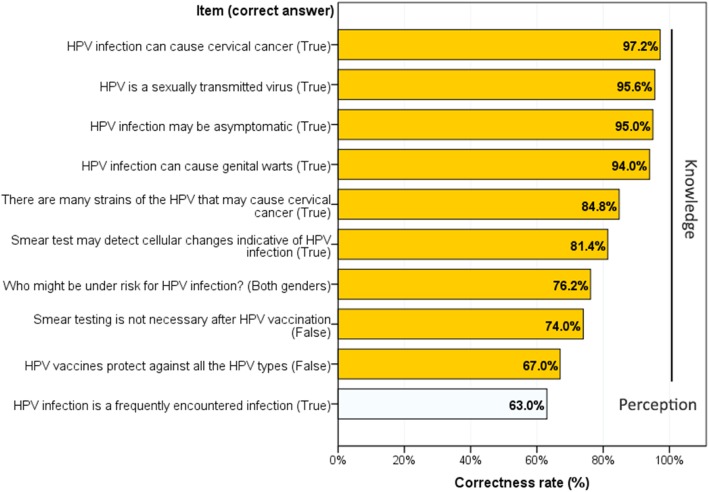
Knowledge and perception among Saudi physicians about human papilloma virus (HPV) infection and vaccine. Bars represent the percentage of physicians who answered correctly to the given item

While majority of the participants replied correctly to all knowledge questions, knowledge about HPV-related risk of cervical cancer (97.2%), sexual transmission mode (95.6%), and whether HPV infection may be asymptomatic (95.0%) totalized the highest correctness rates. The lowest correctness rate was observed for the item whether HPV vaccines protect against all HOP serotypes (67.0%). Further, 63.0% of the participants perceived HPV infection as a frequently encountered infection (Fig. 1). Regarding knowledge score, descriptive statistics showed mean = 7.65, SD = 1.45, median = 8, range = 2–9; consequently, 62.0% of the participants had adequate knowledge (score ≥ 8) (Results not presented).

### Factors and predictors of knowledge

Both nonparametric tests analyzing knowledge score and chi-square test analyzing the percentage of adequate knowledge level showed statistically significant differences in all demographic and professional factors (Table 2). These showed higher knowledge levels among females, non-Saudi, widowed and married, senior consultants and Obstetrics-Gynecologists compared to their counterparts. Regarding age, younger (20–30 years old) participants had the lowest scores and totalized the lowest percentage of adequate knowledge. Regarding region, physicians from Central region (Riyadh) had the highest score (mean = 8.12 versus < 8) and percentage of adequate level (83.1% versus < 65%) compared to other regions.

**Table 2:** Factors associated with knowledge about HPV infection and vaccine

Parameter / category	N	Knowledge score				Adequate knowledge (score ≥ 8)		
		Mean	Median	Range	*p*-value^1^	Freq.	%^§^	*p*-value^2^
Gender								
Female	1056	7.86	8	2–9		724	68.6	
Male	944	7.41	8	2–9	<.001*	516	54.7	<.001*
Nationality								
Saudi	1340	7.50	8	2–9		772	57.6	
Others	660	7.96	8	2–9	<.001*	468	70.9	<.001*
Age groups								
20–30	352	7.17	7	2–9		160	45.5	
30–40	608	7.82	8	3–9		392	64.5	
40–50	508	7.87	8	3–9		364	71.7	
50–60	420	7.55	8	2–9		252	60.0	
Above 60	112	7.64	8	3–9	<.001*	72	64.3	<.001*
Marital status								
Single	268	7.39	7	3–9		132	49.3	
Married	1588	7.69	8	2–9		1012	63.7	
Widowed	20	8.20	8	7–9		16	80.0	
Divorced	124	7.68	8	4–9	<.001*	80	64.5	<.001*
Practice level								
Resident	500	7.26	7	2–9		240	48.0	
Junior	496	7.75	8	3–9		332	66.9	
Senior	1004	7.80	8	2–9	<.001*	668	66.5	<.001*
Specialty								
Ob/gyn	1172	8.17	9	2–9		916	78.2	
Non ob/gyn	818	6.92	7	2–9	<.001*	324	39.1	<.001*
Region								
Western	1148	7.54	8	2–9		660	57.5	
Eastern	308	7.58	8	2–9		188	61.0	
North	116	7.97	8	6–9		72	62.1	
South	116	7.37	8	3–9		64	53.3	
Central	308	8.12	8	2–9	<.001*	256	83.1	<.001*

^§^Percentages are calculated on the factor categories. Ob/gyn: Obstetrics-gynecology; * statistically significant result (*p* < 0.05); test used: ^1^ nonparametric tests (Mann-Whitney U test and Kruskal-Wallis test, as appropriate), ^2^ chi-square test

The multivariate model including all these factors showed that inadequate knowledge (score < 8) was independently associated with the physicians’ nationality (Saudi: OR = 1.51, *p* = 0.003), practice level (resident: OR = 3.53, *p* < 0.001; junior OR = 1.67, *p* = 0.002), specialty (non Ob-Gyne: OR = 5.40, *p* < 0.001), and region (Western: OR = 2.54, Eastern: 3.22, Northern: 4.32 and Southern: 5.55); as well as age (Table 3).

**Table 3:** Independent factors of inadequate knowledge about HPV infection and vaccine

Predictor / category	OR	95%CI		*p*-value
Gender				
Female	*Ref*	–	–	–
Male	1.18	0.94	1.47	.151
Nationality				
Saudi	1.51	1.15	1.99	.003*
Others	*Ref*	–	–	–
Age groups				
20–30	*Ref*	–	–	.005*
30–40	1.22	0.82	1.83	.333
40–50	1.35	0.83	2.20	.228
50–60	2.08	1.25	3.44	.005*
Above 60	2.30	1.20	4.42	.012*
Marital status				
Single	1.42	0.84	2.42	.194
Married	1.14	0.73	1.79	.564
Widowed	0.40	0.11	1.41	.155
Divorced	*Ref*	–	–	.166
Level of practice				
Resident	3.53	2.35	5.33	<.001*
Junior	1.67	1.21	2.30	.002*
Senior	*Ref*	–	–	–
Specialty				
*Ob/gyn*	*Ref*	–	–	–
Non ob/gyn	5.40	4.31	6.76	<.001*
Region				
Western	2.54	1.78	3.63	<.001*
Eastern	3.22	2.13	4.87	<.001*
Northern	4.32	2.54	7.37	<.001*
Southern	5.55	3.29	9.36	<.001*
Central	*Ref*	–	–	–

Binary logistic regression; dependent variable: inadequate knowledge; OR: odd ratio; CI: confidence interval; *Ref*: category used as reference to calculate OR; * statistically significant result (*p* < 0.05)

### Opinions and attitude regarding HPV vaccine

A minority of the participating physicians declared being already vaccinated against HPV (7.6%) and less than half opined that they agree to be vaccinated (41.2%); however, majority were favorable to vaccinate their children or own children (77.6%) and to include HPV vaccine in the local immunization program (69.6%). Reasons for HPV vaccine refusal among participants who disagreed to be or to have their children vaccinated (*N* = 1196) included the following: not being under the risk of HPV infection (58.5%); lack of knowledge about the vaccine (21.1%); being sexually inactive (14.7%); vaccine not being reimbursed by the government (8.7%); and fear from vaccine side effects (8.4%); while 10.0% had no reason for refusing (Table 4).

**Table 4:** Opinions and attitudes regarding HPV vaccination among Saudi physicians

Parameter / category	Number	Percentage
Attitudes (*N* = 2000)		
Already received HPV vaccine	152	7.6
Agree to be vaccinated	824	41.2
Agree to vaccinate own/future children	1552	77.6
Favorable to including the HPV vaccine in the Saudi immunization program	1392	69.6
Reasons for refusal (*N* = 1196, 59.8%)^a^		
Not under risk for HPV infection	700	58.5
Lack of knowledge about HPV vaccine	252	21.1
Vaccine has many side effects	100	8.4
Vaccine not reimbursed	104	8.7
Not sexually active	176	14.7
Do not know	120	10.0

^a^Refusal was defined as disagreement by the participant to be vaccinated or to have his/her children/future children vaccinated (*N* = 1196); and percentage for each reason was calculated on refusers. More than one reason may be provided by a participant

### Factors associated with opinions and attitudes toward HPV vaccine

The rate of HPV immunization was higher among younger (12.5%) followed by older (10.7%) physicians (*p* < 0.001), single (10.4%) followed by married (7.6%) ones (*p* = 0.044), those from Ob/Gyn specialty (8.9%, *p* = 0.011) and those from the Western regions (10.1%, *p* < 0.001) compared to their counterparts. Additionally, those who perceived HPV infection as being frequent had higher rate of immunization (8.6% versus 5.9%) compared to their counterparts (*p* = 0.032). On the other hand, no association with knowledge level was found (*p* = 0.317). Other opinion and attitude parameters showed statistically significant associations with all or almost all demographic factors. Most notably, overall negative attitude regarding HPV vaccination was typically associated with male, older, Saudi, senior consultant in other than Ob/Gyn specialty. Additionally, participants with inadequate knowledge level were less favorable to vaccinate their children (67.9% versus 83.5%, *p* < 0.001) and to inclusion of the vaccine in the local immunization program (58.9% versus 76.1%, *p* < 0.001). Interestingly, participants who perceived HPV infection as being prevalent were remarkably more favorable to vaccinate their children (82.9% versus 68.2%, *p* < 0.001) and to inclusion of HPV vaccine in the Saudi immunization program (76.5% versus 57.8%, *p* < 0.001) (Table 5).

**Table 5:** Factors associated with attitude regarding HPV vaccination

Factor	Category	Already received HPV vaccine		Agree to be vaccinated		Agree to vaccinate own/future children		Favorable to include HPV vaccine in Saudi program	
		%	*p*-value	%	*p*-value	%	*p*-value	%	*p*-value
Gender	Female	8.3		48.9		79.5		71.2	
	Male	6.8	.191	32.6	<.001*	75.4	.027*	67.8	.001*
Nationality	Saudi	7.5		42.4		75.5		67.2	
	Non-Saudi	7.9	.741	38.8	.124	81.8	.001*	74.5	<.001*
Age groups	20–30	12.5		54.5		83.0		70.5	
	30–40	7.2		51.3		83.6		72.4	
	40–50	6.3		40.9		76.4		72.4	
	50–60	4.8		23.8		68.6		66.7	
	Above 60	10.7	.001*	10.7	<.001*	67.9	<.001*	50.0	<.001*
Marital status	Single	10.4		55.1		82.1		71.6	
	Married	7.6		40.1		78.1		70.0	
	Widowed	0.0		0.0		80.0		60.0	
	Divorced	3.2	.044*	32.3	<.001*	61.3	<.001*	61.3	<.001*
Level of practice	Resident	8.8		63.2		85.6		75.2	
	Junior	5.6		34.7		75.8		66.9	
	Senior	8.0	.141	33.5	<.001*	74.5	<.001*	68.1	<.001*
Specialty	Ob/gyn	8.9		44.0		82.6		77.8	
	Non Ob/gyn	5.8	.011*	37.2	.002*	70.5	<.001*	58.0	<.001*
Region	Western	10.1		39.7		77.4		67.9	
	Eastern	1.3		37.7		71.4		61.0	
	North	6.9		55.2		75.9		75.9	
	South	3.3		43.3		66.7		76.7	
	Central	6.5	<.001*	44.2	.010*	89.6	<.001*	79.2	<.001*
Knowledge level	Adequate	8.1		41.3		83.5		76.1	
	Inadequate	6.8	.317	41.1	.917	67.9	<.001*	58.9	<.001*
Perception about HPV	True	8.6		41.3		82.9		76.5	
	False	5.9	.032*	41.1	.934	68.6	<.001*	57.8	<.001*

Percentages are calculated on the factor categories. Oby/gyn: Obstetrics-gynecology; * statistically significant result (*p* < 0.05); test used: chi-square

## Discussion

### Summary of findings

Improving awareness and enhancing pro-HPV vaccination attitude among physicians constitute the cornerstone of successful prevention programs against HPV infection and cervical cancer epidemics. This nationwide survey puts another brick in the wall and paves the way for a national systematic vaccination program that is slow to appear. It showed that majority of physicians working in Saudi Arabia have adequate knowledge about HPV infection and vaccination, as demonstrated by the high mean and median knowledge score and more than 60% having adequate knowledge level. On the other hand, a minority declared being already immunized (7.6%), at the time of the survey, or favorable to receive the vaccine (41.2%), which contrasted with the majority being favorable to vaccinate their children (77.6%) and to include the vaccine in the local immunization program (69.6%). The combination of these observations suggest that physicians are partially aware about the imminent threat of HPV infection on the Saudi population, expecting a shift to an endemic trend of the disease. This emerging awareness was more perceptible among young and female physicians, as well as among non-Saudi and particularly among Ob/Gyn specialists. This issues the hypothesis of an increasing number of cases that are diagnosed in the clinical practice, by these categories of physicians. Further, findings of this study enabled profiling the typical physicians with certain reluctance to systematic vaccination against HPV.

### Adequate knowledge to improve physicians’ attitude regarding vaccination

A few studies have assessed the level of physicians’ knowledge about HPV infections and vaccines in conservative or Middle Eastern communities as Saudi Arabia. A recent study (2018) among Saudi primary health care physicians showed high knowledge scores about both HPV infection and vaccine, along with positive attitude regarding the necessity of the vaccine. However, only 16.5% declared routinely providing vaccine recommendation to their patients; and this was associated with higher knowledge score and better perception about the necessity of the vaccine [1]. An earlier investigation (2011) among Saudi physicians regarding their practice in cervical cancer screening showed that half of the respondents only were favorable to recommend the vaccine to their patients, in addition to frequent misconceptions regarding cervical cancer [22]. Regionally, a study from Kuwait showed relatively high knowledge levels about cervical cancer causal relationship with HPV, other risk factors, and clinical presentation. As well, moderate to high rates of knowledge were observed regarding cervical cancer screening and HPV infection pathogenesis and detection methods. Similar to our findings, majority of the Kuwaiti physicians (75%) were favorable to systematic vaccination of schoolgirls [26].

Comparable observations were reported in international literature. For example, a Mexican nationwide study demonstrated high levels of awareness among physicians about HPV-cervix cancer causal relationship with significantly better knowledge among Ob-Gyn specialists than GPs, notably regarding the pathogenic mechanisms of this relationship. The same study demonstrated favorable opinions among the physicians regarding the pertinence of educating women about the causal relationship of cervical cancer with HPV; however, a significant proportion opined that such information might cause disruptions between partners, confusions or unnecessary anxiety among the population [27]. In India, relatively lower levels of knowledge were reported among both community and academic physicians, with less than half the participants being aware of the availability of HPV vaccine locally. Furthermore, a minority (30%) declared being favorable to recommend HPV vaccination to their patients, which was predicted by the lack of knowledge about the long-term efficacy of the vaccine [28].

Studies that were carried out among other healthcare providers showed comparable results. An Indian study involving healthcare providers from 232 hospitals and 80 PHCs demonstrated that less than half respondents recommended HPV vaccine to their patients although majority had adequate knowledge about the causal relationship with cervical cancer and were aware about the importance of the vaccine [29]. In Turkey, both nurses and nursing students showed high-to-acceptable levels of theoretical knowledge about HPV infection and its contribution in cervical cancer, as well as about the vaccine availability. However, this knowledge contrasted with marginal immunization rate (< 2%), minority complying with regular screening, and only one-third being favorable to receive the vaccine [30–32]. Contrastingly, a Nigerian study showed lower levels of knowledge and awareness about HPV vaccine among nurses, while a significant number were favorable to be vaccinated and supported vaccination of preadolescent girls [33]. Another Indian study among medical and paramedical students reported lower knowledge levels about HPV infection and vaccination, notably regarding vaccine efficacy [34]. In Italy, nursing students had high levels of awareness and knowledge about HPV infection and its relationship with cervical cancer, while lower knowledge was noted regarding risk factors and the efficacy of the HPV vaccine in preventing cervical cancer. On the other hand, a higher immunization rate (24%) was reported, almost 90% of them were females, and two-third of the non-vaccinated ones were favorable to receive the vaccine. Further, approximately 92% of these Italian nursing students exhibited willingness to recommend the vaccine to their future students, and such attitude was independently associated with knowledge about the risk factors of HPV infection and the efficacy of the vaccine in preventing cervical cancer [35].

Regardless of using different tools and methods to assess knowledge and opinions, conclusions from the previous studies converge towards two major issues: 1) a generally poor engagement among physicians and health providers in HPV vaccination among their patients despite a good theoretical knowledge; 2) a positive effect of knowledge on such engagement. This indicates that increasing knowledge and awareness among the physicians about HPV infection and vaccination is crucial to enhance a positive attitude, but not sufficient to obtain active and efficient engagement in the clinical practice. Further, the combination of the previously cited data suggests that the efficacy of the vaccine in preventing cervical cancer may be a powerful argument to convince physicians to promote the vaccine among their patients and the society.

### Barriers to vaccination: to vaccinate vs to be vaccinated

The other determining factor of physicians’ engagement in promoting HPV vaccine among their patients may be the presence of eventual barriers that stand between the theoretical knowledge and practice. In the present study, participants raised a number of reasons to explain their refusal to receive the vaccine or to have their children vaccinated. These included lowly perceived risk for HPV infection, lack of knowledge about the vaccine and concerns about the vaccine safety and cost. Similar to our findings, previously cited Indian study among medical and paramedical students showed that almost half of the respondents were reluctant to receive the vaccine, presenting concerns about side effects and cost of the vaccine or declaring being underexposed to the risk of contagion [34]. Likewise, concerns about the vaccine safety were reported by majority of Italian nursing students, and positive attitude regarding vaccine safety was predicted by knowledge about HPV risk factors and vaccine efficacy in preventing cervical cancer. Other reasons behind refusal to be vaccinated among this population included uncertainty about the vaccine efficacy, fear of eventual adverse effects, and lowly perceived risk of contracting the infection [35]. Other barriers were reported in an Indian study that investigated the factors associated with the intention to recommend HPV vaccine, where respondents worried about patients’ refusal of the vaccine and raised societal and cultural barriers that may impede communication with the parents of adolescent children; and these worries were remarkably shared by Ob/Gyn specialists [36]. In the present study, the presence of such obstacles was associated with 17–53% less positive opinions about systematization of the HPV vaccine (Results not presented). Furthermore, physicians who refused to be vaccinated (OR = 1.51, *p* < 0.001) or to have their children vaccinated (OR = 3.17, *p* < 0.001) had significantly higher propensity to refuse including HPV vaccine in the Saudi immunization program (Results not presented). Consequently, the presence of such barriers among physicians constitute not only barriers to accept the vaccine at the individual level, but indicate a general adverse attitude towards HPV vaccine that could downplay the physicians’ role in promoting the vaccine among patients. Therefore, identifying and alleviating these obstacles is the second most important step in physician’s education in HPV vaccination.

Further, the issue of vaccine cost and its reimbursement by the government appear to be of significant weight on doctors’ attitude in the routine practice. A study from Hong Kong, showed that two-third primary health care physicians involved in vaccination program for schoolgirls were favorable to HPV vaccine becoming fully paid by the government, stressing that this would considerably enhance the acceptance of the vaccine and the coverage rate among the population [37].

### The need for targeted educational interventions

The present study highlighted male, older, Saudi, and senior consultants in specialties other than Ob/Gyn as the typical physician profile associated with adverse opinions regarding HPV vaccination and a presumably lower propensity to take part in vaccination campaigns and to promote HPV vaccination among patients and the population. Similar observations were reported locally, where better attitude regarding the vaccine was evidenced among female and younger PHC physicians, while male and older ones had comparably poorer perception of the importance of HPV vaccine [1]. Such age and gender difference may be justified by females being more sensitive to cervical cancer issue and by a generation effect on the perception of the HPV epidemic and threat to the society. The observation about age may be supported by the paradoxical findings regarding the level of experience, which showed a higher acceptance rate among residents and junior doctors to receive the vaccine, to vaccinate their own children and to include the vaccine in the national program despite lower levels of knowledge compared to seniors. This may denote a transformation in the medical training content, providing more attention to HPV infections and the importance of immunization to prevent cervical cancer. Regarding nationality, Saudi physicians had lesser knowledge scores and less favorable attitude regarding the inclusion of HPV vaccine in the Saudi immunization program, compared to non-Saudi ones. This is probably due to a lower perception of the extent of HPV epidemics and, consequently, a lower sense of urgency regarding vaccination given the conservative background [38–40]. Regarding specialty, this study showed lower levels of knowledge among physicians from specialties other than Ob/Gyn along, along with more adverse attitude towards vaccinations as demonstrated by lower acceptance rates to receive the vaccine and to have own children vaccinated and less favorability to include the vaccine in the national program. Such findings were expectable owing to more advanced education on HPV infections and cervical cancer in the Ob/Gyn curriculum, besides the greater clinical exposure to such cases. Differences between specialties were also observed by Almughais et al., who reported greater knowledge scores among Saudi family medicine physicians by comparison to their peers from other specialties, which was explained by larger contact with the public community, while other specialties examine patients upon medical referrals [1]. Therefore, physicians with such “adverse profiles” should be the target of more intensive educational interventions, which should aim at fixing the misconceptions about the HPV vaccine and updating the physician’s roles in a changing society that is increasingly exposed to sexually transmitted diseases.

### Limitations of the study

The major strengths of this study was its sampling methodology including large sample size and physicians’ recruitment from the five regions of the country. However, it had some methodological issues that may limit the generalization of its findings. First, the cross-sectional design does not enable establishing a causal relationship between physicians’ levels of knowledge and opinions and attitude regarding the vaccine and vaccination program. Second, only physicians working in public sector were included, while those from private sector or academic filed were not included, which may have a significant impact on knowledge level as well as in vaccination and vaccine recommendation practice. Third, the questionnaire included basic questions and did not probe into advanced issues, which may result in overestimation of the knowledge level. Additionally, the questionnaire failed to explore the physicians’ practice in recommending the vaccine to their patients.

Despite these limitations, findings from this study contribute in characterizing the unmet needs in physicians’ education regarding HPV infection and vaccination and shed light on some barriers and misconceptions that may hinder the physicians’ engagement in promoting the vaccine. It highlights efficient and targeted educational interventions among physicians as being the next crucial step for a successful prevention strategy against HPV infections and cervical cancer. On the other hand, the Saudi government and health authorities should tackle the ambient challenges including the societal and cultural barriers by means of effective communication strategies to break the apprehensiveness wall between physicians and patients, as well as a minimum subsidy to overcome vaccine cost among the most eligible groups of the population.

## Conclusions

Majority of physicians from the Saudi public sector have adequate basic knowledge about HPV infection and vaccination, which was associated with more positive attitude regarding vaccine. A generational shift in opinions and attitude regarding HPV vaccine was observed, denoting an emerging awareness about the HPV threat to the society among younger physicians.

On the other hand, lowly perceived risk for HPV infection, lack of knowledge about the vaccine and concerns about the vaccine safety and cost were frequently reported as reasons to refuse the vaccine by the physicians and were indicative of a general adverse attitude towards HPV vaccine that could downplay the physicians’ role in promoting the vaccine among patients.

Therefore, more specific educational interventions are warranted to trigger an active engagement among physicians in the fight against HPV infection and cervical cancer. Such interventions should demystify the HPV vaccine by exposing its efficacy, availability and safety, along with providing practical information about the vaccination procedure, local policies and goals to achieve via the vaccination and screening campaigns. Additionally, exposing an accurate epidemiological picture that highlights the imminent threat of the HPV infection to the Saudi society would raise greater awareness about the necessity of the vaccine.

Finally, the present study highlighted male, older, Saudi, and senior consultants in specialties other than Ob/Gyn as the typical physician profile associated with adverse opinions regarding HPV vaccination and a presumably lower propensity to take part in vaccination campaigns and to promote HPV vaccination among patients and the population. Therefore, physicians with such “adverse profiles” should be the target of more intensive educational interventions, which should aim at fixing the misconceptions about the HPV vaccine and updating the physician’s roles in a changing society that is increasingly exposed to sexually transmitted diseases.

## Additional file

**Additional file 1.** Physician’ Knowledge and Opinions on Human Papillomavirus Vaccination: A Cross-Sectional Study from Saudi Arabia.

## Data Availability

Data is available from the corresponding author upon request.

## Abbreviations

HPVs: Human papilloma viruses
MNG-HA: Ministry of National Guard Health Affairs
MOH: Ministry of Health
PHC: Primary health care
SD: Standard deviation
SPSS: Statistical Package for Social Science
