# Supplementary material for: Physician’s knowledge and opinions on human papillomavirus vaccination: a cross-sectional study, Saudi Arabia
Source: BMC Health Serv Res. 2019 Dec 12;19:963. doi: 10.1186/s12913-019-4756-z (PMC6909584; doi:10.1186/s12913-019-4756-z)
Supplement: Supplementary file 1 — Additional file 1. Physician’ Knowledge and Opinions on Human Papillomavirus Vaccination: A Cross-Sectional Study from Saudi Arabia. [file 12913_2019_4756_MOESM1_ESM.docx]

**Physician’ Knowledge and Opinions on Human Papillomavirus Vaccination: A Cross-Sectional Study from Saudi Arabia.**

| 1. Gender:   *Female * Male |
| --- |
| 2. Nationality  * Saudi * Non Saudi |
| 3. Age:  *20-30 * 31-40 * 41-50 * 51-60 *61-70 |
| 4. Marital Status:  * Single * Married * Widowed * Divorced |
| 5. What is your Practice level?  * Resident * Registrar * Consultant |
| 6. What is your specialty?  * Obstetric and Gynecology  * Family medicine  * Pediatric  * Dentist  * Medicine  * Surgical |
| 7. Which Region are you Practice?  * Western Region  * Eastern Region  * North Region  * Southern Region  * Central Region |
| Please Pick an Answer for the Questions Below to The Best of your Knowledge. |
| 8. Human papillomavirus infection is a frequently encountered infection.  * True * False * I don't know |
| 9. Human papillomavirus is a sexually transmitted virus.  * True * False * I don't know |
| 10. Human papillomavirus infection may be asymptomatic.  * True * False * I don't know |
| 11. Who might be under risk for Human papillomavirus infection?  * Women * Men * Both |
| 12. Human papillomavirus infection can cause genital warts.  * True * False * I don't know |
| 13. Human papillomavirus infection can cause cervical cancer.  * True * False * I don't know |
| 14.There are many strains of the Human papillomavirus virus that may cause cervical cancer. * True * False * I don't know |
| 15. Human papillomavirus vaccines protect against all the HPV types.  * True * False * I don't know |
| 16. Smear testing is not necessary after human papillomavirus vaccination  * True * False * I don't know |
| 17.The smear test may detect cellular changes indicative of a Human papillomavirus infection * True * False * I don't know |
| 18. Did you have a Human Papillomavirus Vaccine?  * Yes * No |
| 19. Do you Want to be Vaccinated?  * Yes * No |
| 20. Do you want your children /future children to be Vaccinated?  * Yes * No |
| 21. What is/are your reason for human papillomavirus vaccine refusal?  *I'm not under risk for a human papillomavirus infection  *Human papillomavirus vaccine is not a commonly administered vaccine. I don't have knowledge regarding it  *The vaccine has many side effects  *The government does not pay for the vaccine cost  *I'm not sexually active  *I don't know  *I'm not with vaccine refusal |
| 22. There are many countries in the world in which Human papillomavirus vaccination is part of a school age immunization program. Do you think including the vaccine in the Saudi immunization program is necessary?  * Yes * No *I don't know or I don't care |

**QUESTION 21 CAN CHOOSE MORE THAN ONE ANSWERS**
